# Successful treatment of *Talaromyces marneffei* pneumonia in a HIV-negative renal transplantation recipient: A case report

**DOI:** 10.1097/MD.0000000000030958

**Published:** 2022-10-07

**Authors:** De-Han Cai, Jun Wang, Xiao-Lin Fang

**Affiliations:** a Nephrology Department in Jiangxi Provincial People’s Hospital Affiliated to Nanchang Medical College, Nanchang, Jiangxi, China; b Department II of Respiratory and Critical Care in Jiangxi Provincial People’s Hospital Affiliated to Nanchang Medical College, Nanchang, Jiangxi, China.

**Keywords:** mNGS, renal transplantation, tacrolimus, *Talaromyces marneffei*, voriconazole

## Abstract

*Talaromyces marneffei* causes life-threatening opportunistic fungal infections in immunocompromised patients. It often has a poorer prognosis in non-human immunodeficiency virus (HIV)-infected than in HIV-infected individuals because of delayed diagnosis and improper treatment.

**Patient concerns:**

A 51-year-old man presented with complaints of pyrexia, cough, and expectoration that had lasted for 15 day. This patient has been taking anti-rejection medication since kidney transplant in 2011.

**Diagnosis:**

*T marneffei* pneumonia; post renal transplantation; renal insufficiency; hypertension.

**Interventions:**

Intravenous moxifloxacin was administered on admission. After the etiology was established, moxifloxacin was discontinued and replaced with voriconazole. The tacrolimus dose was adjusted based on the blood concentration of tacrolimus and voriconazole.

**Outcomes:**

The patient was successfully treated and followed-up without recurrence for 1 year.

**Lessons:**

A high degree of caution should be maintained for the possibility of *T marneffei* infection in immunodeficient non-HIV patients who live in or have traveled to *T marneffei* endemic areas. Early diagnosis and appropriate treatment can prevent progression of *T marneffei* infection and achieve a cure. Metagenomic next-generation sequencing (mNGS) can aid the physician in reaching an early pathogenic diagnosis. Close monitoring of tacrolimus and voriconazole blood levels during treatment remains a practical approach at this time.

## 1. Introduction

*Talaromyces marneffei* is responsible for invasive fungal infections, with the highest prevalence in Southeast Asia and southern China.^[1]^ It can cause both local and disseminated infections. The latter are more common, involve the monocyte-macrophage system, and often cause pneumonia before spreading to the liver, spleen bone marrow, lymph nodes, skin, and other organs via lymph and blood circulation. The symptoms reflect the corresponding multi-organ damage. With an increasing occurrence in human immunodeficiency virus (HIV)-negative hosts, talaromycosis has emerged as a serious public health concern.^[2]^

This case of *T marneffei* pneumonia occurred in an HIV-negative kidney transplantation patient.

## 2. Case report

A 51-year-old man was admitted to our hospital on November 30, 2020 with complaints of pyrexia, cough, and expectoration that had lasted for 15 day. Two weeks before admission, the patient had experienced night sweats and fever below 37.5 ºC without cough, stuffy or runny nose, or other respiratory symptoms. He had sought treatment at a local hospital but his symptoms did not improve after 10 day of intravenous administration of piperacillin-tazobactam and azithromycin; in addition, he developed a cough with yellow, purulent sputum.

The patient had been taking immunosuppressants following a kidney transplantation that had been carried out in 2011 for renal failure. His most recent medications were mycophenolate mofetil (750 mg qd; 1000 mg qn), tacrolimus (1 mg qd; 0.5 mg qn), and prednisone (5 mg, qd). His most recently recorded creatinine levels showed maintenance between 145 µmol/L and 175 µmol/L before admission. The patient denied a history of chronic diseases, such as hypertension or diabetes, as well as of infectious diseases, such as tuberculosis or hepatitis.

The patient had been living in Lushan City in Jiangxi Province, southern China, with no history of tobacco or alcohol abuse and no relevant family history. He was a cadre and usually worked in an office.

Physical examination at admission showed body temperature of 37.9 °C, respiration of 20 beats/minute, heart rate of 90 beats/minute, and blood pressure of 145/78 mm Hg. There was no enlargement of superficial lymph nodes throughout the body. There were no skin lesions nor joint abnormalities. A few wet rales could be heard in the right lower lung. The liver and spleen were not felt under the ribs on abdominal palpation.

Routine blood tests revealed a normal white cell count of 9.22 × 10^9^/L, high neutrophil count of 80% (normal range: 50%–70%), low hemoglobin of 90 g/L (normal range: 120–165 g/L), and a normal platelet count. Hypoproteinemia (26.9 g/L; normal range: 35–50 g/L) and elevated creatinine (175 µmol/L; normal range: 50–110 µmol/L), blood 1-3-β-D-Glucan (437.67 pg/mL; normal range <20 pg/mL), blood galactomannan (2.8 pg/mL, normal range <0.5 pg/mL), and C-reactive protein (67 mg/L; normal range: 5–10 mg/L) were observed. Liver function, electrolytes, arterial blood gases, procalcitonin, T-spot tuberculosis test, HIV antibody, and blood cryptococcus capsular antigen levels were all normal or negative. Blood T cell subsets included low lymphocytes of 12.33% (normal range: 27.9%–37.3%), high T lymphocytes of 96.48% (normal range: 62.6%–76.8%), normal CD4 + T cells of 618.48/µL, and low CD8 + T cells of 351.23/µL (404–754/µL).

After the patient was transferred to our hospital, intravenous moxifloxacin was administered and the mycophenolate mofetil dose was decreased to 250 mg, twice daily.

**Figure 1.**
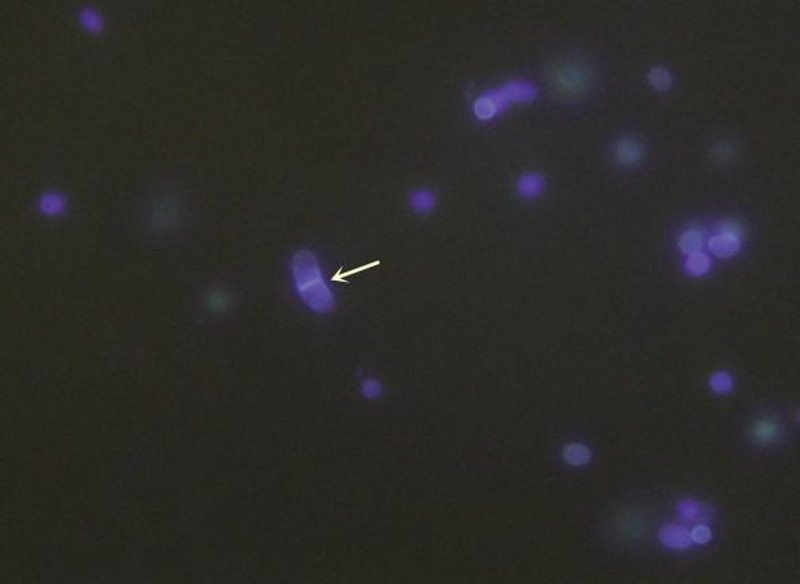
Fluorescence staining of a bronchoalveolar lavage fluid smear showing sausage-like spores with a central septum (white arrow). Magnification: ×400.

**Figure 2.**
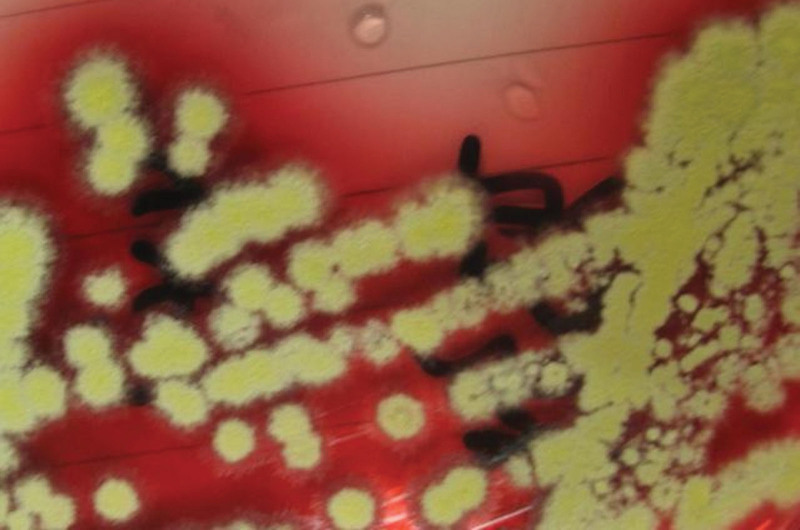
Yellow villous colonies producing water soluble wine pigment appeared after incubation at 26 ºC.

**Figure 3.**
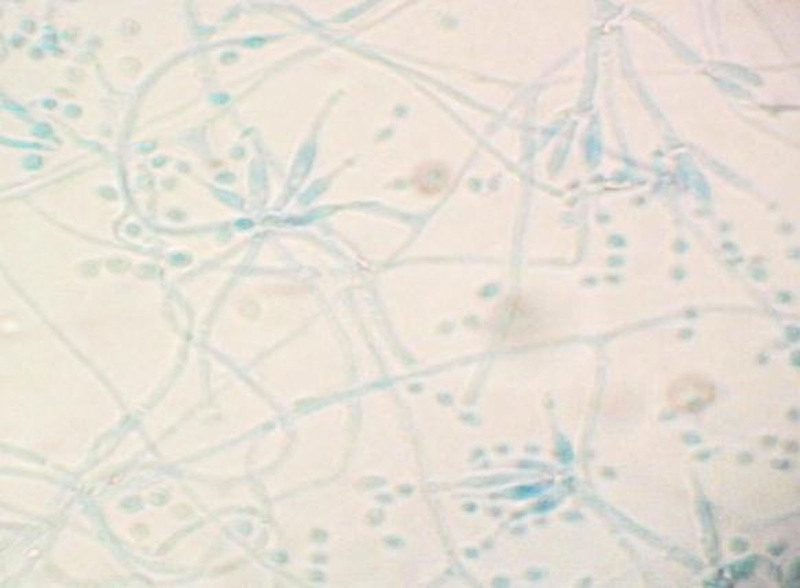
Brush-shaped hyphae were visible by light microscopy in a smear preparation of colony culture at 26 ºC.

On December 2, 2020, bronchoscopy revealed yellow, purulent sputum at the opening of the right lower lobe. After sputum aspiration, narrow openings were found in the basal trunk and dorsal segment; the mucosa was hyperemic and hypertrophic. On December 3, 2020, fluorescent staining of a bronchioalveolar lavage fluid (BALF) smear found sausage-like spores with a central septum (Fig. 1). On December 4, 2020, metagenomic next-generation sequencing (mNGS) of a BALF specimen detected *T marneffei* (2339 reads, 98.15% cover rate), and a 7-day culture of BALF yielded biphasic *T marneffei* (Figs. 2 and 3).

**Figure 4.**
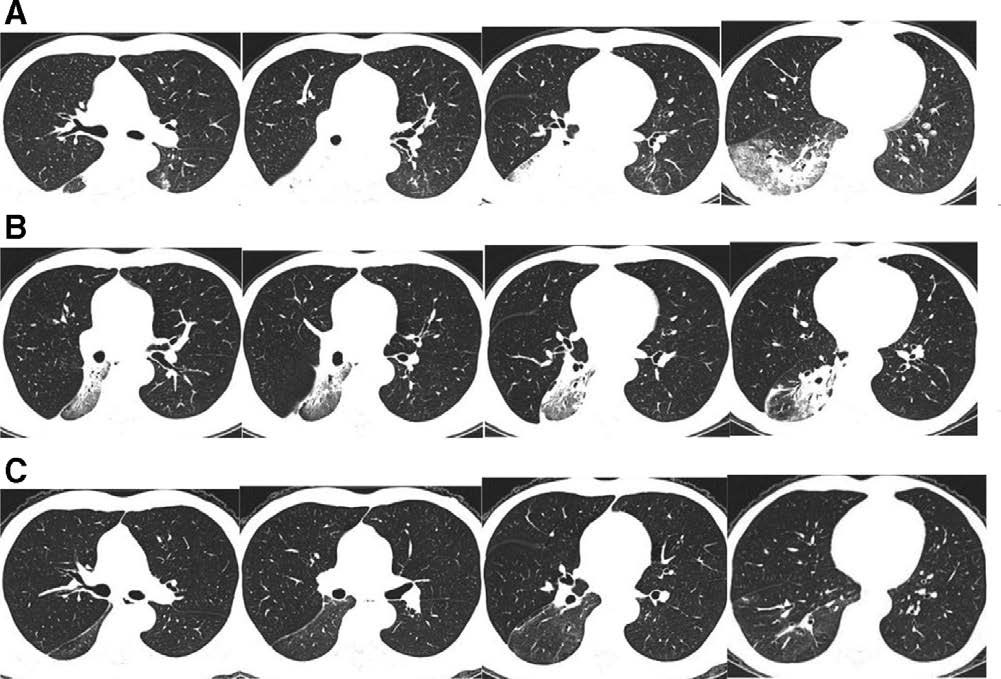
Computed tomography. A: There was a large consolidation in the dorsal segment of the right lower lobe, patchy exudation with partial consolidation in the basal segment of the right lower lobe, and right lower hilar lymphadenopathy (December 7, 2020); B: The lesion was obviously absorbed (January 4, 2021); C: The lesion was almost completely absorbed with few fibrous cords remaining (February 15, 2021).

Chest computed tomography (CT) at the local hospital showed a solid lesion in the right lower lung. A repeat chest CT at our hospital showed an enlarged lesion (Fig. 4A).

We did not find other disseminated lesions after a thorough evaluation, including of the abdomen, brain, lymph nodes, skin, and joints. The patient was diagnosed with *T marneffei* pneumonia.

**Figure 5.**
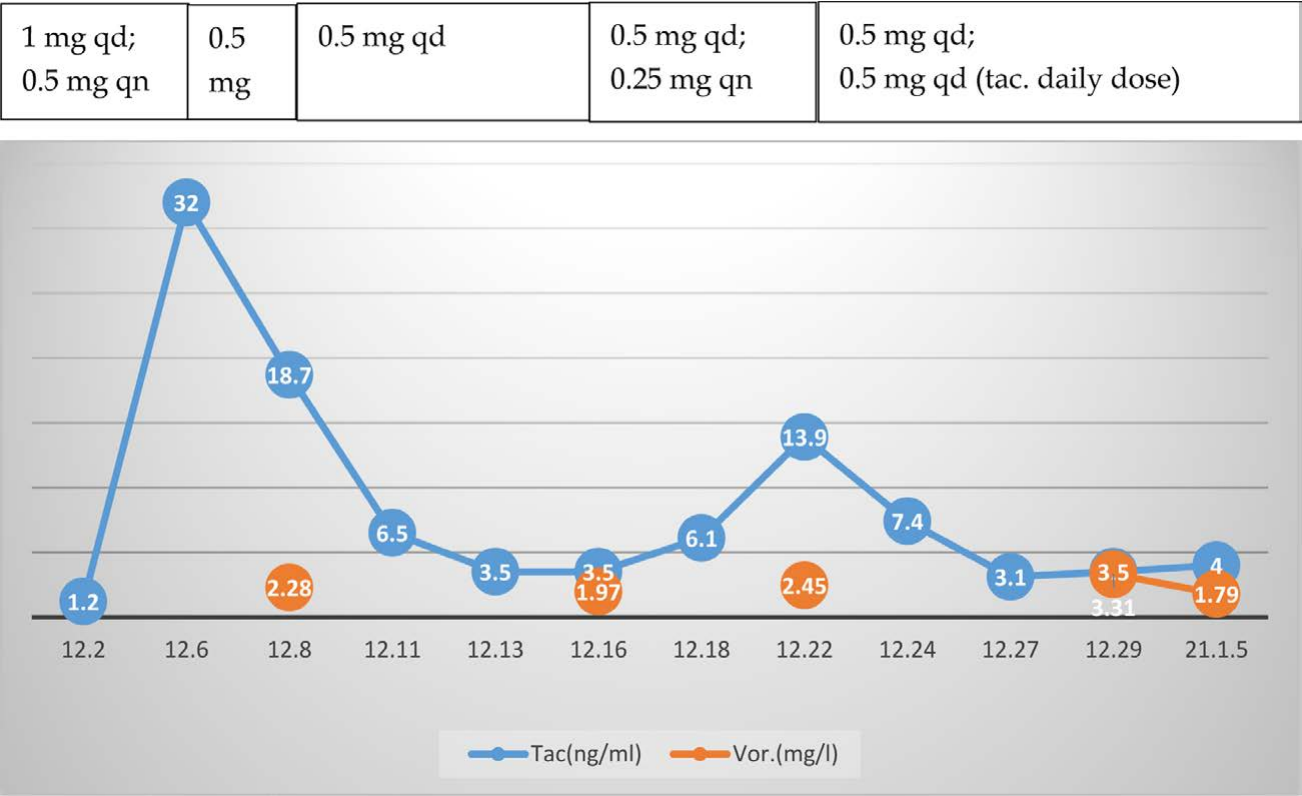
Blood concentration of tacrolimus and voriconazole with tacrolimus daily dose adjustment. tac. = tacrolimus; vor. = voriconazole.

On December 4, 2020, moxifloxacin was discontinued and replaced with voriconazole (200 mg, po, bid) because the patient’s creatinine clearance was less than 50 mL/min. The tacrolimus dose was adjusted frequently, especially in the first month, to maintain the blood tacrolimus and voriconazole concentrations within the proper concentration range (Fig. 5). The lung lesion had absorbed on February 15, 2021, and voriconazole was discontinued after 3 month of oral administration (Fig. 4B, C). Patient follow-up of 1 year did not find evidence of recurrence.

## 3. Discussion

*T marneffei* can cause opportunistic invasive fungal infections, with high rates of recurrence and mortality. It is the third leading cause of opportunistic infections in acquired immune deficiency syndrome (AIDS) patients, following tuberculosis and cryptococcus.^[3]^ Highly active antiretroviral therapy has led to a decline in HIV-related *T marneffei* infections, while infections in HIV-negative patients have increased. The comorbidities present in patients with *T marneffei* infections include malignancies, organ transplantation, autoimmune diseases, and some emerging conditions such as adult-onset immunodeficiency associated with anti-interferon gamma antibodies, T lymphocyte-depleting immunosuppressive drugs, and novel targeted anticancer agents such as anti-CD20 monoclonal antibodies and kinase inhibitors. Because of its insidious onset and rapid progression, the diagnosis of talaromycosis is often delayed, which leads to high fatality rate. Furthermore, talaromycosis mortality is higher in HIV-negative than in HIV-positive patients.**^[2]^** Early diagnosis of talaromycosis is both crucial and challenging.

*T marneffei* is the only thermally dimorphic penicillium, with a mold phase at 25 to 28 ºC and producing a diffusible wine-red pigment and brush-shaped microscopic hyphae microscopically, and a nonpigmented pathogenic yeast phase pigment at 37 ºC. Confirmation of talaromycosis requires the isolation of biphasic penicillium from specimen cultures.

In this case, the clinician communicated with the microbiology technologist to suspect a fungal possibility due to the patient’s immunocompromised condition. After fluorescence staining revealed spores with transverse septa, the possibility of *T marneffei* was considered. Hence, the specimen was incubated at both 26 and 37 ºC. It was not until the 7th day after the lavage fluid specimen was collected that it was confirmed that the causative organism of the patient’s infection was indeed *T marneffei*. Hien et al indicated that the culture may even take up to 14 days for identification.**^[4]^** In comparison, the actual testing time for mNGS was only 24 hours, excluding the logistical time for specimen delivery. It was this valuable time lag that allowed clinicians to quickly target the pathogen and adjust the treatment plan in time so that the disease did not spread further.

In addition to the time consuming nature of culture, its positive rate is not as good as it could be. Liu et al confirmed a higher positive rate for BALF mNGS (64%) than for BALF culture (28%).**^[5]^** Li et al**^[6]^** found that mNGS had a diagnostic sensitivity of 88.89% and specificity of 74.07% with an agreement rate of 77.78% compared to specimen culture. Compared with culture smears and polymerase chain reaction, mNGS had a diagnostic sensitivity of 77.78% and a specificity of 70.00%.

Because of the long time, low accuracy and low positive rate of traditional culture, it often leads to delayed diagnosis and inappropriate use of antibiotics. Although mNGS has some inadequacies, such as interference with human-derived nucleic acids, report interpretation, and high cost, it is still increasingly used in clinical practice because of its high throughput, high timeliness, high accuracy, and broad coverage. Furthermore, BALF-based mNGS is recommended for diagnosing pulmonary fungal infections due to its diagnostic advantages over conventional tests.**^[7]^**

In this case, fluorescent staining of BALF smears suggested that the pathogen was *T marneffei*. Fluorescent dyes that specifically bind to chitin and dextran in the fungal cell wall can be used to label fungi, to allow observation of cellular morphology by fluorescence microscopy. Because it is rapid, economical and direct, fluorescence staining is now widely used in clinical practice.**^[8]^**

As the patient was found to have renal insufficiency before the onset of infection, oral voriconazole was chosen. When voriconazole is used in combination with tacrolimus, which is also metabolized by CYP3A, the blood concentration of tacrolimus could quickly rise to very high levels.**^[9,10]^** The drug manufacturer suggested that the concurrent tacrolimus dose be reduced by one-third; other case reports described an 80% to 90% reduction.**^[10,11]^** One report noted that the voriconazole dose was also reduced as appropriate.**^[12]^**

The optimal therapeutic windows for tacrolimus in patients with renal transplants are 5 to 15 µg/L at 1 to 3 month after surgery and 3 to 8 µg/L beginning at 12 month after surgery.**^[13]^** Nevertheless, because of individual differences, genotype polymorphisms, and differences of pharmacokinetics and clinical backgrounds, there are no consistent criteria or recommendations for adjusting blood tacrolimus concentration in patients with invasive fungal infection after organ transplantation. Fortunately, in our case, with intensive monitoring, the tacrolimus dose was adjusted to maintain a blood concentration of 3 to 6 ng/mL. The voriconazole blood concentration also remained within the effective range (1.79–3.31 mg/L; normal range: 1–5.5 mg/L). The patient was finally cured and renal function was maintained at the pre-onset level.

In conclusion, clinicians should be alert to the possibility of *T marneffei* infection in HIV-negative immunocompromised patients with a history of tourism or residence in *T marneffei* endemic regions. mNGS can powerfully assist physicians in quickly and accurately targeting the pathogen, preventing further spread and worsening of the infection. Fluorescent staining is a diagnostic hint for suspected fungal infections. Individual differences and narrow treatment windows for tacrolimus require close monitoring of its blood concentration during the administration of antifungal treatment with voriconazole or other azole drugs.

## Acknowledgments

The authors are grateful to Dr Hui Chen for providing photos of the microorganism.

## Author contributions

Fang X-L conceived the study and developed the search strategy, supervised the patient’s diagnosis and treatment during the entire process, and put forward valuable opinions, and edited the manuscript; Cai D-H and Wang J conducted the literature review and participated in the patient’s diagnosis and treatment; Cai D-H produced the draft of the manuscript; All authors contributed to the final manuscript.

**Conceptualization:** Xiao-Lin Fang.

**Formal analysis:** Xiao-Lin Fang.

**Methodology:** Jun Wang.

**Writing – original draft:** De-Han Cai.

**Writing – review & editing:** Xiao-Lin Fang.
